# The landscape of immune cell infiltration in the glomerulus of diabetic nephropathy: evidence based on bioinformatics

**DOI:** 10.1186/s12882-022-02906-4

**Published:** 2022-09-05

**Authors:** Wei ZHOU, Yaoyu LIU, Qinghong Hu, Jiuyao ZHOU, Hua LIN

**Affiliations:** 1 Department of Clinical Pharmacy, The Second Affiliated Hospital, Guangzhou University of Chinese Medicine, Guangzhou, Guangdong People’s Republic of China; 2 Department of Pharmacology, School of Pharmaceutical Sciences, Guangzhou University of Chinese Medicine, Guangzhou, Guangdong People’s Republic of China

**Keywords:** Diabetic nephropathy, Immune cell infiltration, CIBERSORT, Bioinformatics, GSEA (gene set enrichment analysis)

## Abstract

**Background:**

Increasing evidence suggests that immune cell infiltration contributes to the pathogenesis and progression of diabetic nephropathy (DN). We aim to unveil the immune infiltration pattern in the glomerulus of DN and provide potential targets for immunotherapy.

**Methods:**

Infiltrating percentage of 22 types of immune cell in the glomerulus tissues were estimated by the CIBERSORT algorithm based on three transcriptome datasets mined from the GEO database. Differentially expressed genes (DEGs) were identified by the “limma” package. Then immune-related DEGs were identified by intersecting DEGs with immune-related genes (downloaded from Immport database). The protein–protein interactions of Immune-related DEGs were explored using the STRING database and visualized by Cytoscape. The enrichment analyses for KEGG pathways and GO terms were carried out by the gene set enrichment analysis (GSEA) method.

**Results:**

11 types of immune cell were revealed to be significantly altered in the glomerulus tissues of DN (Up: B cells memory, T cells gamma delta, NK cells activated, Macrophages.M1, Macrophages M2, Dendritic cells resting, Mast cells resting; Down: B cells naive, NK cells resting, Mast cells activated, Neutrophils). Several pathways related to immune, autophagy and metabolic process were significantly activated. Moreover, 6 hub genes with a medium to strong correlation with renal function (eGFR) were identified (SERPINA3, LTF, C3, PTGDS, EGF and ALB).

**Conclusion:**

In the glomerulus of DN, the immune infiltration pattern changed significantly. A complicated and tightly regulated network of immune cells exists in the pathological of DN. The hub genes identified here will facilitate the development of immunotherapy.

**Supplementary Information:**

The online version contains supplementary material available at 10.1186/s12882-022-02906-4.

## Background

Diabetic nephropathy (DN) is an extremely common complication of diabetes mellitus, affecting up to 30% of individuals with type 1 diabetes mellitus and 40% of individuals with type 2 diabetes mellitus [1]. Over the last two decades, no new drugs have been approved to specifically prevent DN or to improve kidney function [2]. Current strategies to treat this disease mainly focus on intensification control of glycemic, blood pressure and other risk factors [3]. But a significant proportion of patients still progress to end stage renal failure overtime with a well control of risk factors, which highlight the urgent need to identify effective therapies.

Recently, DN is increasingly considered as an inflammatory disease, with immune modulation being involved in every stage. Immune cells infiltrated in kidney tissues, such as macrophages, T cells, and mast cells produce various inflammatory cytokines, metalloproteinases, and growth factors, which modulate the local response and increase inflammation [4–8].

Although there is substantial evidence for the vital roles of macrophages, T cells, B cells, and mast cells, the effect of other immune cells including neutrophils, NK cells, and dendritic cells are less clear. Furthermore, the immune system contains various types of immune cell, which constitute a complex regulatory network, potential intercorrelations of them in the pathological process of DN are less clear. Therefore, an integrated analysis of immune infiltration, differentially expressed genes, and aberrant pathways activation in kidney tissues is essential.

Here, we used the CIBERSORT algorithm to analyze the abundance of 22 kinds of immune cells in the glomerulus samples of DN based on three microarray datasets mined from the Gene Expression Omnibus (GEO) [9]. Then an integrated bioinformatics analysis was conducted to screen immune-related differentially expressed genes(IR-DEGs)and explore aberrant pathways activation. Besides, we evaluated the correlation between IR-DEGs with immune infiltration cells and clinical indicator. Our research provides a potential value for immunotherapy of DN.

## Materials and methods

### Data acquisition

We systematically searched publicly available DN gene expression datasets on Gene Expression Omnibus (GEO) database using the following terms: (“DN” or “DKD” or “diabetic nephropathy” or “diabetic kidney disease”) and “Homo sapiens” [porgn:_txid9606] and (“Expression profiling by array”[Filter]).

**Fig. 1:**
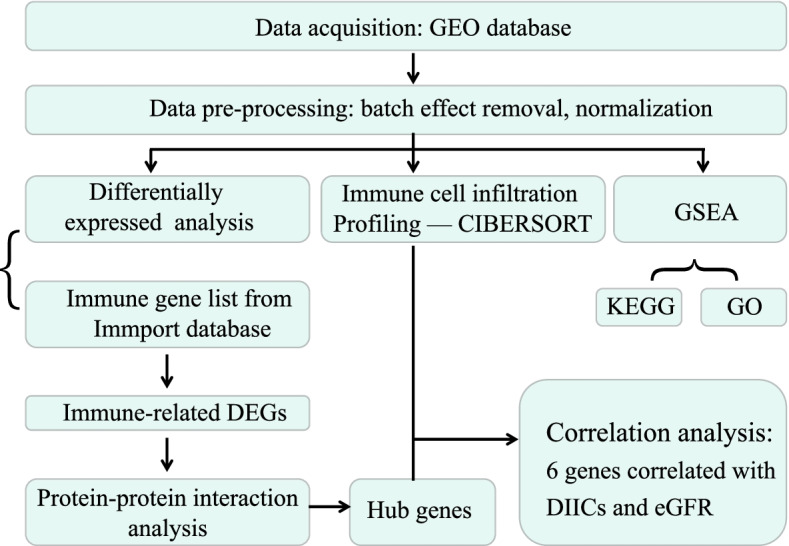
Flowchart of this study. DEGs: differentially expressed genes; CIBERSORT: cell-type identification by estimating relative subsets of RNA transcripts; DIICs: differentially infiltrated immune cells; GO: Gene Ontology; KEGG: Kyoto Encyclopedia of Genes and Genomes; GSEA: Gene set enrichment analysis; eGFR: estimated glomerular filtration rate

Datasets included in this study have to meet the following criteria: (1) The organism of samples is Homo sapiens. (2) The sample type is glomerular tissue. (3) gene expression was detected by microarray. (4) dataset including DN and NC (negative control) samples (5) The dataset contains more than 3 samples per group. Finally, three datasets were included in this research (GSE96804, GSE30528, and GSE47183). Database searches were conducted in February 2022. The detailed workflow of this study is shown in Fig. 1.

### Data pre-processing

The probe ID was converted to a gene symbol using the platform annotation files. If a gene matched multiple probes, the maximum value of multiple probes expression was applied. To avoid sample heterogeneity arising from different technical platforms and dissimilar methods of processing data, batch effect removal and normalization were conducted by the “limma” package [10, 11]. Then Principal component analysis (PCA) was performed to Confirm homogeneity after data pre-processing.

### Immune cell infiltration profiling

A deconvolution algorithm—CIBERSORT—was used to estimate the relative proportion of 22 types of infiltrating immune cell in glomerular tissue by identifying 547 immune cell-related genes [9].

The normalized gene expression data were uploaded to the CIBERSORT web portal (http://CIBERSORT.stanford.edu/) with a parameter of 1000 permutation and a relative mode. *P* < 0.05 was considered to be statistically significant. The Pearson correlation coefficient between each immune cell was calculated and visualized with a correlation heatmap by the “corrplot” package.

### Differentially expressed gene analysis

The “limma” package was used to reveal the differentially expressed genes (DEGs) between DN and NC samples with a threshold of |log2FC (fold change) |> 1 and adjusted *P*-value < 0.05 [11]. The visualization of DEGs was conducted by the “ComplexHeatmap” and “ggplot2” packages.

### Gene set enrichment analysis (GSEA)

The enrichment analyses for Gene Ontology (GO) terms and Kyoto Encyclopaedia of Genes and Genomes (KEGG) pathways were carried out by the gene set enrichment analysis (GSEA) method with the “clusterProfiler” package. 

### Protein–protein interaction (PPI) analysis of immune-related DEGs

The immune-related gene list was downloaded from the ImmPort Database (https://www.immport.org/shared/home). Then immune-related differentially expressed genes (Immune-related DEGs) were identified by intersecting DEGs with immune-related genes. The protein–protein interactions of Immune-related DEGs were estimated by the Search Tool for the Retrieval of Interacting Genes (STRING) database (http://string-db.org). Then genes with a confidence score of interaction more than 0.95 were visualized by Cytoscape (version 3.8.2). The “Cytohubba” plug-in was used to identify the highest linkage hub genes in the network and the parameters used were as follows: the top 10 nodes, ranked by degree. [12].

### Correlation analysis of Hub genes with infiltrated immune cells and clinical indicator

Correlation analysis between hub genes and immune cells were calculated and visualized with the “corrplot” package. Then the hub genes which has a medium to strong correlation with immune cells were conducted correlation analysis with renal function indicator—estimated Glomerular Filtration Rate (eGFR), and visualized with the “ggpubr” package.The eGFR and corresponding gene expression matrix used here were mined from the Nephroseq database (includes transcriptome data of 9 DN patients and 13 NC volunteers glomerular tissue).

### Statistical analysis

All statistical analysis was performed on R software (version 4.0.2). An independent t-test was performed to compare continuous variables between two groups, *p*-value < 0.05 was considered statistically significant. Correlations were assessed by the Pearson correlation coefficient (0.1 <| r |< 0.4 represents a weak correlation; 0.4 <| r |< 0.7 represents a medium correlation; and | r |> 0.7 represents a strong correlation).

## Results

### Data integration and removal of batch effects

First, the GEO series of GSE96804 (41 DN and 20 Negative Controls—NC samples), GSE30528 (9 DN and 13 NC samples), and GSE47183 (14 DN and 14 NC samples) were acquired from the GEO database and merged as one big dataset with a total of 111 samples (64 DN and 47 NC samples). The second series (GSE30528) employs excellent Negative Controls (donor biopsies), while the NC samples of the other two come from the unaffected portion of tumor nephrectomies.

**Fig. 2:**
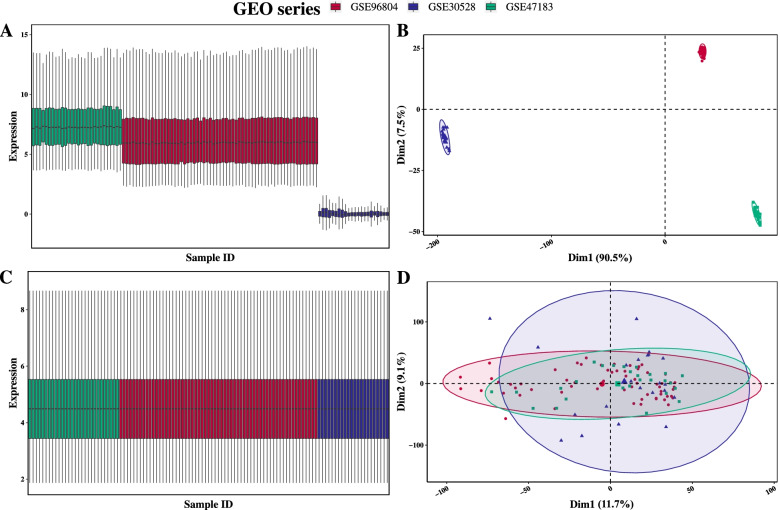
Batch effect removement and data normalization. (**A**-**B**) Box and PCA plot of three GEO series before removal of batch effect and data normalization. (**C**-**D**) Box and PCA plot of three GEO series after removal of batch effect and data normalization

Then, the batch effect was evaluated and visualized by a box plot and a PCA score plot (Fig. 2A-B). Then batch effect removement and data normalization were conducted with the “limma” package. At last, the homogeneity between GEO series was confirmed (Fig. 2C-D).

### Infiltration proportion of immune cells changed in DN group

**Fig. 3:**
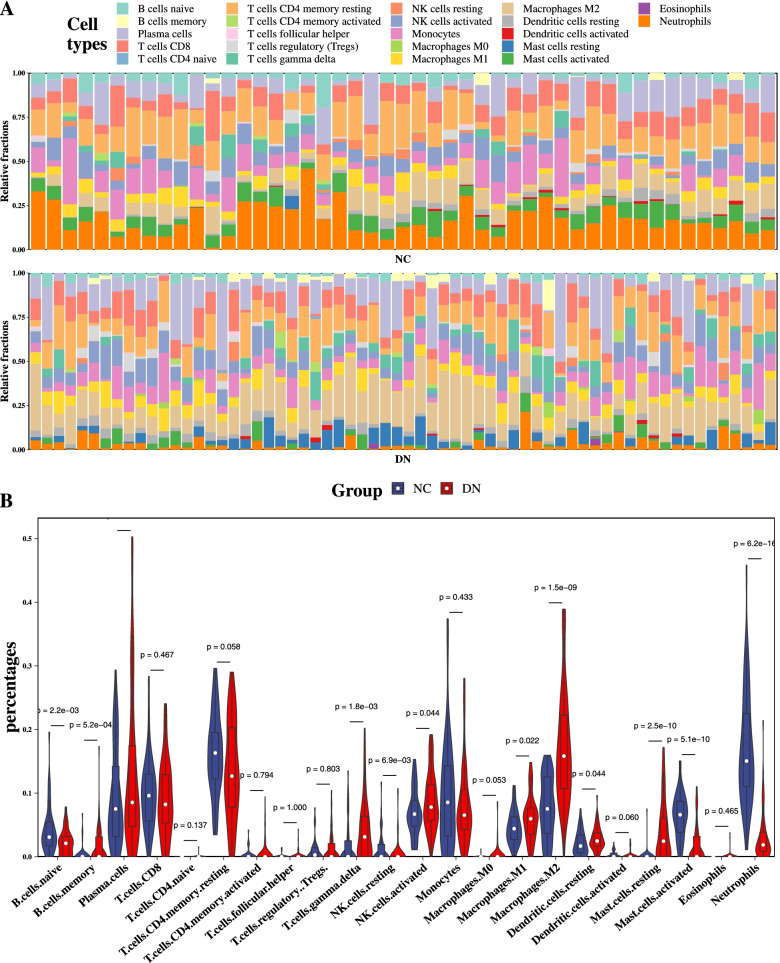
Analysis of immune cell infiltration by the CIBERSORT algorithm. (**A**) Stacked bar plot shows the relative percent of 22 types of immune cell in each sample of two groups. (**B**) Violin plot shows the differences of immune infiltration between two groups

The proportion of 22 types of immune cells in the glomerulus tissues were estimated by the CIBERSORT algorithm. As shown in Fig. 3A, the fraction of immune cells varied significantly among the samples and groups. Due to the limitations of the CIBERSORT algorithm, the infiltration of some low abundance immune cell subsets wasn’t fully revealed. The violin plot shows that 11 types of cells changed significantly in DN. Among which, 7 types were up-regulated and 4 types were down-regulated (Fig. 3B). These results demonstrated that there were different infiltration patterns of immune cells in DN glomerular tissues.

**Fig. 4:**
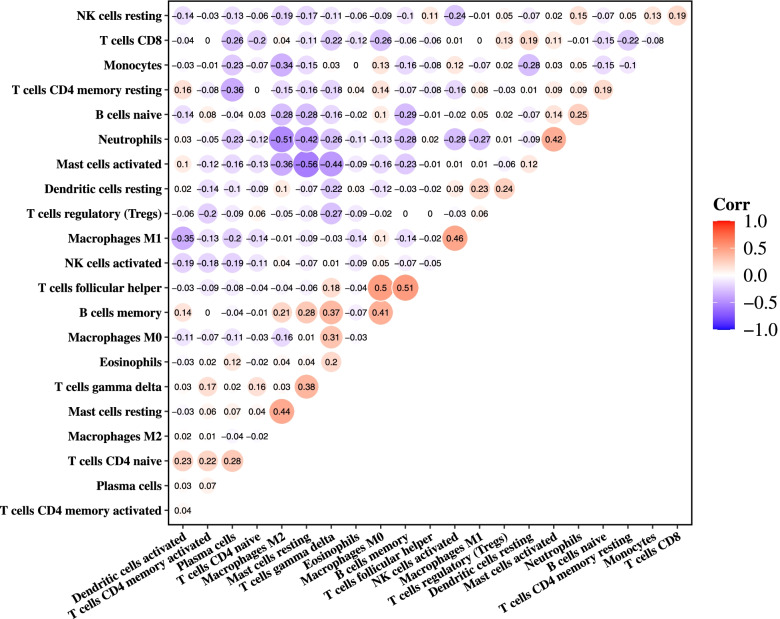
Correlation analysis of 22 types of immune cells. Red: positive correlation; blue: negative correlation

To evaluate whether different types of immune cells interact with each other, correlation analysis was conducted. And we found multiple pairs of positively or negatively related immune cells. Among them, T cells follicular helper and B cells memory show the most synergistic effect (*r* = 0.51, *p* < 0.001); meanwhile, Mast cells activated and Mast cells resting show the most competitive effect (*r* =—0.56, *p* < 0.001) (Fig. 4).

### Identification of differentially expressed genes(DEGs)

**Fig. 5:**
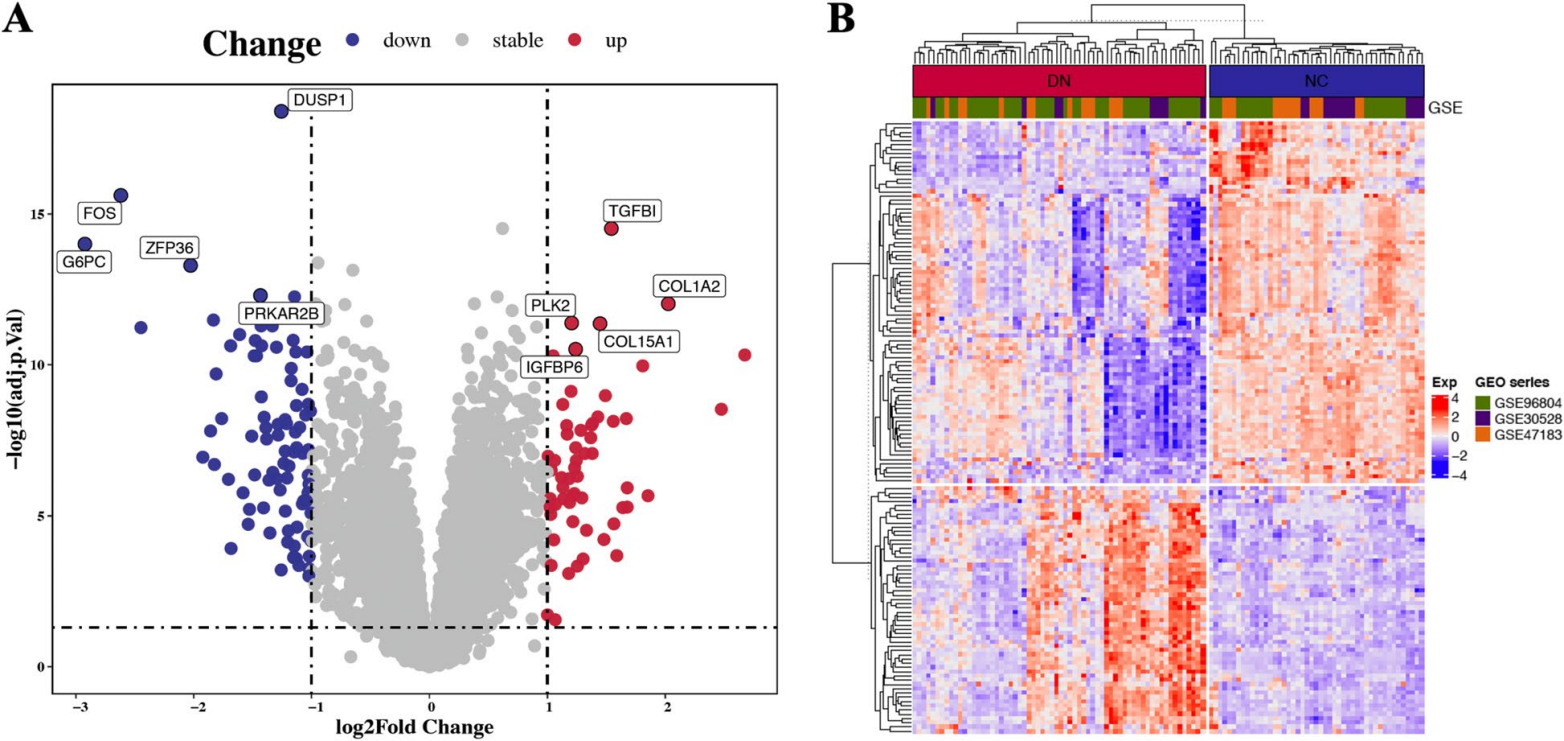
Differential gene expression analysis for DN vs. NC. (**A**) Volcano plot of dysregulated genes (|log2FC (fold change) |> 1 and adjusted *P*-value < 0.05). Blue points represent relatively downregulated genes, red points represent upregulated genes, gray points represent genes showing no significant alteration. (**B**) Heatmap of the 143 DEGs expression level among 111 samples

In differential expression analysis (64 DN vs. 47 NC), a total of 143 differentially expressed genes were identified, of which 58 were upregulated and 85 were downregulated in DN. To visualize the DEGs more intuitively, we plotted a volcano plot and a heatmap plot (Fig. 5A-B). The expression details of the top 10 upregulated and top 10 downregulated genes are listed in Table 1.

**Table1:** The top 10 upregulated and top 10 downregulated genes identified by differential gene expression analysis

Symbol	log_2_FC	AveExpr	p.Value	adj.P.Val	Change
DUSP1	-1.25617	5.29114	3.76E-23	4.01E-19	down
FOS	-2.6175	4.872139	4.56E-20	2.44E-16	down
TGFBI	1.542813	5.209413	1.14E-18	3.06E-15	up
G6PC	-2.92274	4.707322	4.68E-18	9.99E-15	down
ZFP36	-2.02578	6.488748	3.35E-17	5.1E-14	down
PRKAR2B	-1.43332	6.333434	4.26E-16	5.06E-13	down
C1orf21	-1.14408	4.950416	5.25E-16	5.61E-13	down
COL1A2	2.025326	5.13844	1.24E-15	9.49E-13	up
PDK4	-1.83224	5.740396	5.58E-15	3.3E-12	down
PLK2	1.206103	5.42454	8.49E-15	4.12E-12	up
COL15A1	1.446617	4.378624	9.28E-15	4.31E-12	up
LPL	-1.42772	4.903369	1.16E-14	5.14E-12	down
CA10	-1.33274	5.008615	1.2E-14	5.14E-12	down
ALB	-2.44695	4.936927	1.48E-14	5.86E-12	down
IGFBP6	1.239681	5.188632	1.17E-13	3.05E-11	up
LUM	2.673499	5.665852	2.12E-13	4.72E-11	up
GPR18	1.048852	3.723417	2.58E-13	5.04E-11	up
COL6A3	1.808742	4.569743	6.48E-13	1.08E-10	up
MMP2	1.200659	4.856786	6.22E-12	7.55E-10	up
THBS2	1.491578	4.540541	9.03E-12	1.05E-09	up

### Gene set enrichment analysis (GSEA)

**Fig. 6:**
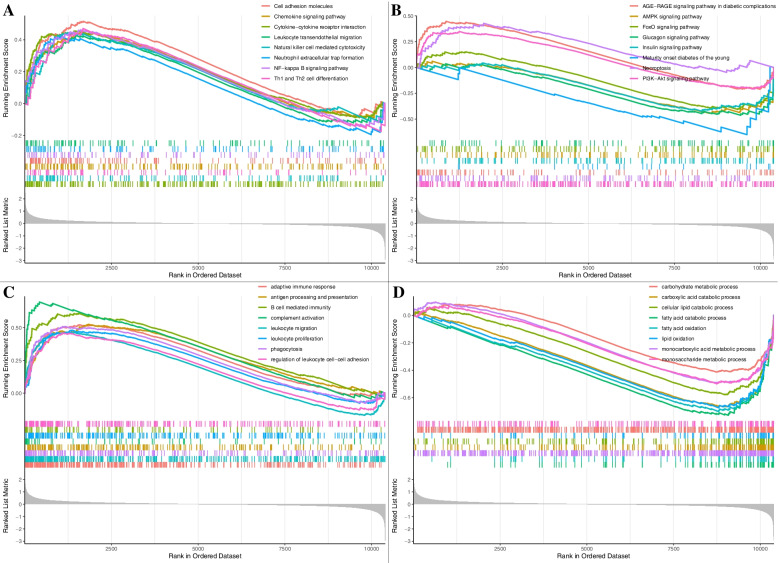
Results of KEGG and GO by the GSEA method. (**A**) 3 representative enriched immune-related KEGG pathways. (**B**) 3 representative enriched DN-related KEGG pathways. (**C**-**D**) 8 representative enriched immune related GO gene sets. GSEA: Gene set enrichment analysis; KEGG: Kyoto Encyclopaedia of Genes and Genomes; GO: gene ontology

To explore the possible pathways and gene sets associated with immune functions, we performed GSEA. Results show that several pathways related to immune, autophagy, diabetes, and Kidney disease were significantly activated in the DN group, including Leukocyte transendothelial migration, Chemokine signaling pathway, FoxO and PI3K − Akt signaling pathway etc. (Fig. 6A-B).

To explore the immune-related gene function in the pathological of DN, GO biological processes (GO-BP) analysis was conducted using GSEA. Eight representative immune-related and eight metabolic-related biological processes were shown in Fig. 6C-D. These results show that immune and metabolic-related processes were activated aberrantly in the glomerulus of DN.

### PPI network construction of immune-related DEGs

**Fig. 7:**
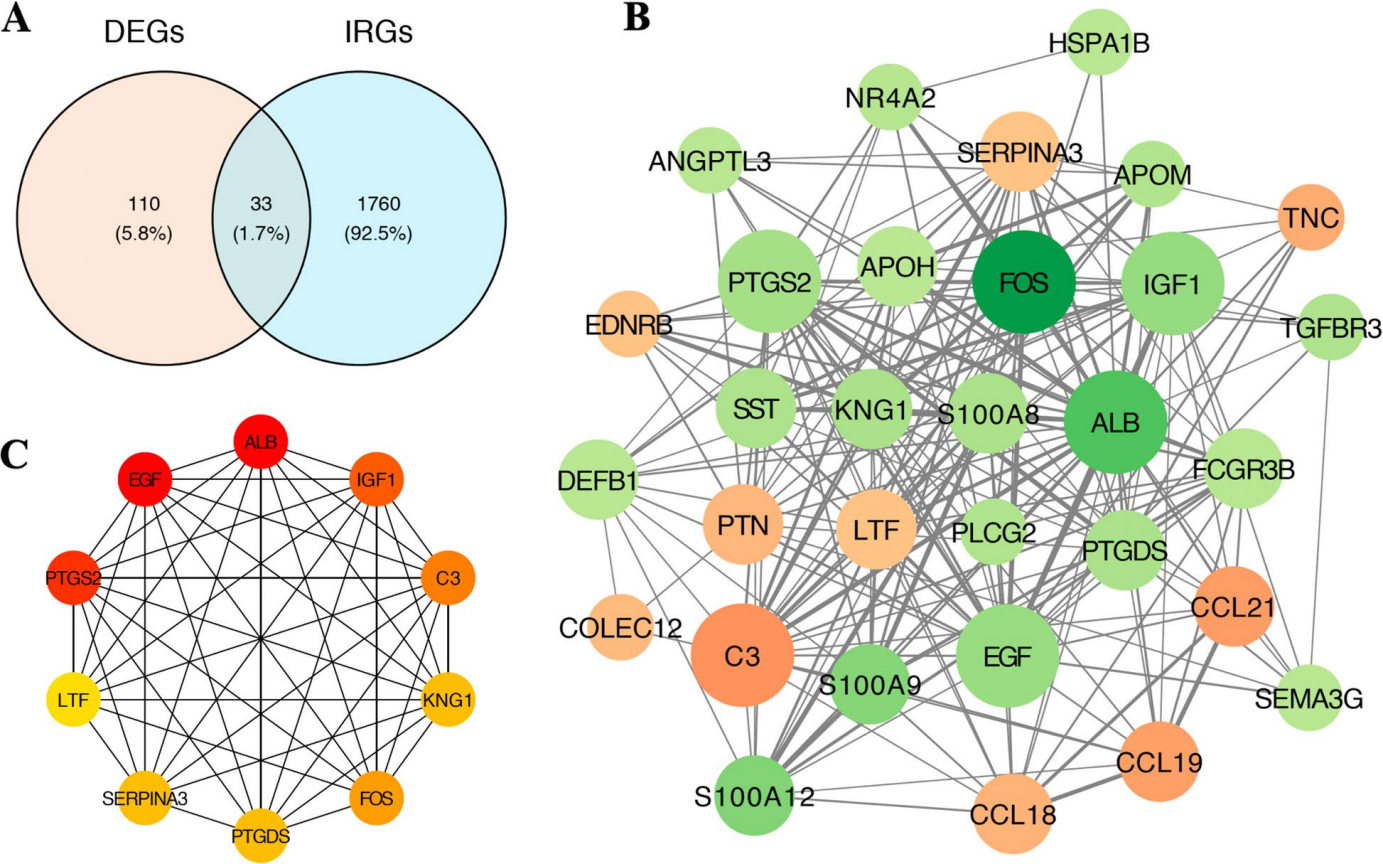
PPI network construction of Immune-related DEGs. (**A**) The intersection of 143 DEGs with 1793 immune-related gene from the Immport database. (**B**) PPI of immune-related DEGs (The node size indicates the clustering coefficient, a larger size indicated a larger clustering coefficient; The node color indicates the log2FC of DEGs, orange represents up regulated genes, while green represents down regulated genes). (**C**) hub gene identified by the degree algorithm using the “Cytohubba” plug-in. IRGs: immune related genes from the Immport database

Thirty-three immune-related differentially expressed genes were obtained by intersecting DEGs with the lists of immune-related genes (IRGs) obtained from the ImmPort database (Fig. 7A). To get a further understanding of the regulation effects among these genes, a PPI (Protein–protein interaction) was constructed by the “Cytoscape” (Fig. 7B), and ten hub genes were identified by the “Cytohubba” plug-in (Fig. 7C).

### Correlation analysis between hub genes and infiltrated immune cells

**Fig. 8:**
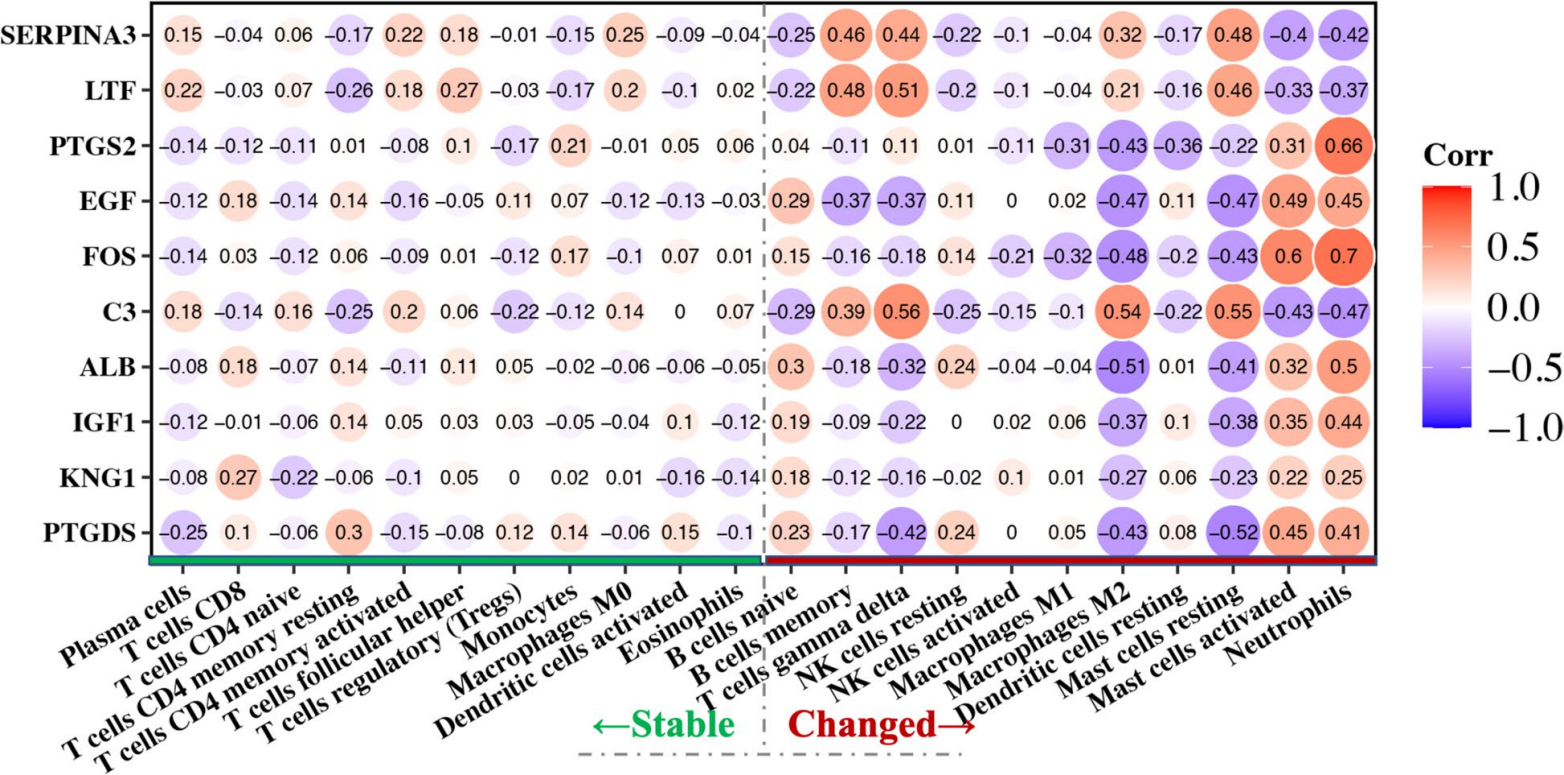
Correlation analysis of hub genes with infiltrated immune cells. (The red line on the bottom right of the picture indicates differentially infiltrated immune cell types in DN, While the line on the bottom left indicates the types with no significant difference)

To understand the molecular mechanisms of alteration in infiltrating immune cells in detail, the correlations between immune related hub genes and immune cells need to be unveiled. So, we calculated Pearson's correlation coefficient for hub genes with immune cells. Figure 8 demonstrates that 9 of the 10 hub genes have moderate to high correlations with differential infiltrated cells(*r* > 0.4), Especially FOS with neutrophils (*r* = 0.7). However, none of the hub genes shows a moderate or higher association with non-differentially infiltrating immune cells. This suggests that these genes may be associated with the retention of immune cells in the glomerulus.

### Exploration of the association between hub genes and clinical indicator

**Fig. 9:**
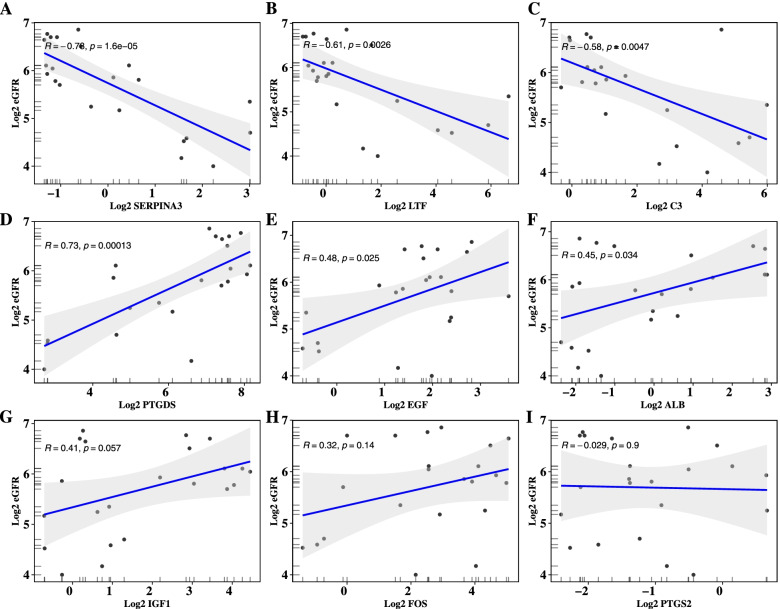
Correlation analysis of hub genes and eGFR

Furthermore, we explored the correlations between the 9 hub genes and the vital clinical renal function indicator—eGFR—based on data mining from the Nephroseq database (version: v5). The data show that 3 genes are negatively correlated with eGFR levels (SERPINA3, LTF, C3), 3 genes are positively correlated with it (PTGDS, EGF, ALB), but the other 3 did not meet the statistical threshold (Fig. 9).

## Discussion

With the advancement of bioinformatics, efficient algorithms have accelerated the transition of various omics big data to new therapeutic targets. As one of the most robust algorithms for analyzing immune cell infiltration—CIBERSORT—has been used in multiple tissues [13–16]. In this study, we used it to explore the landscape of immune infiltration in the glomerulus of DN.

In summary, we found that a variety types of infiltrated immune cell have altered during the pathogenesis of DN. Correlation analysis shows that the immune response may act as a complicated and tightly regulated network. In addition, we also screened out 6 immune-related hub genes which not only have correlations with differently infiltrated immune cells but also have a medium to strong correlation with eGFR (the most important renal function indicators).

Serpin Family A Member 3 (SERPINA3) was revealed to associated with acute immune responses in human renal allograft, and could be a novel biomarker for timely diagnosis [17, 18]. And Andrea Snchez-Navarro found urinary SerpinA3 could detect renal fibrosis and inflammation, with a particular potential for the early detection of AKI to CKD transition [19]. Lactotransferrin (LTF) was reported could be delivered to the Intracerebral hemorrhage affected brain by infiltrating polymorphonuclear neutrophils, and may assist in hematoma detoxification [20]. Qi Zhao found LTF could regulate the immune microenvironment of prostate cancer through JAK/STAT3 Pathway [21]. Nick Wlazlo revealed that Complement C3 (C3) were longitudinally associated with insulin resistance in type 2 diabetes over a 7-year follow-up period, and may reflect progression of metabolic dysregulation [22]. A diabetes control and complications Trial found PTGDS (prostaglandin-H2 D-isomerase), strongly and positively associated with the albumin excretion rate in Type 1 Diabetes Mellitus [23]. Boris B. Betz performed urinary peptidomics analysis in a rodent DN model (Cyp1a1mRen2, renin-dependent hypertension and diabetes mellitus synergized to produce massive albuminuria), and found that urinary epidermal growth factor (EGF) was more than twofold reduced in rats with DN in comparison with controls [24]. Hypochlorite modified albumin (ALB) is an active compound that is formed during the reaction between proteins, and was found to be much higher in concentration and to act as a mediator of oxidative stress and inflammation in patients with uremia or diabetes mellitus [25]. These results show that the 6 hub genes might act as vital roles in the pathological of DN.

It is of interest that neutrophils decreased drastically in DN patients, which is in contrast to the study of an STZ (streptozotocin) induced diabetic mouse model [26]. There are several possible reasons for this opposite conclusion: (1) the impact of intervention drugs may impair neutrophils activity. SCIENCE TRANSLATIONAL MEDICINE reported that angiotensin-converting enzyme inhibitors (ACEI) intervention significantly reduced neutrophils activity compared to treated with an (angiotensin receptor blocker) ARB or with no drug in vitro and in vivo. What's more, ACE knockout neutrophils showed decreased survival signaling and increased apoptosis [27]. (2) the negative feedback regulation of the immune microenvironment; (3) the sample size of this study is still not enough, resulting in abnormal results.

There are some related bioinformatics researches based on DN transcriptome data, but these literatures mainly focus on screening DEGs and exploring related pathways [28–31]. To the best of our knowledge, there is no research report about using transcriptome data to reveal the landscape of immune cell infiltration in DN renal tissue. In addition, the hub genes we reported here were not only highly correlated with renal function, but more importantly, were highly correlated with differentially infiltrated immune cells. In short, we focused on immune infiltration and screened out 6 hub genes may associate with DN immune infiltration.

Nevertheless, some limitations still existed in our study: first, the infiltration of immune cells was estimated by algorithm, rather than based on more direct evidence, such as cell markers. Second, although batch effect removal and normalization were done, the homogeneity of samples between different datasets is still not fully guaranteed. For example, the DN samples may come from patients with very different pathological stages, or with other co-morbidities. And some NC samples come from tumor nephrectomies, which could have changes related to inflammation or alterations in vasculature. So, the results of immune cells differential infiltration stated here is far from definitive. Third, the correlation analysis of hub genes with eGFR is done based on a relatively small sample data (only 22 samples), so this may lead to over- or underestimation of its importance. And the exact mechanism of hub genes involved in immune infiltration had not been investigated through biological experiment in the present study. So, there is an urgent need to conduct in vivo experiments to confirm the results with higher-level evidence.

## Conclusions

In conclusion, our work provides a global picture of the immune environment in the glomerulus of DN. The potential therapeutic targets identified in the present study may provide new insightful for clinical treatment.

## Supplementary Information

**Additional file 1.**

## Data Availability

All raw data are available in GEO datasets (GSE96804, GSE30528, and GSE1009).

## Abbreviations

DN: Diabetic nephropathy
DEGs: Differentially expressed genes
GEO: Gene Expression Omnibus
GO: Gene Ontology
KEGG: Kyoto Encyclopedia of Genes and Genomes
PCA: Principal component analysis
GSEA: Gene set enrichment analysis
STRING: Search Tool for the Retrieval of Interacting Genes
eGFR: Estimated glomerular filtration rate
