# Supplementary material for: The landscape of immune cell infiltration in the glomerulus of diabetic nephropathy: evidence based on bioinformatics
Source: BMC Nephrol. 2022 Sep 5;23:303. doi: 10.1186/s12882-022-02906-4 (PMC9442983; doi:10.1186/s12882-022-02906-4)
Supplement: Supplementary file 1 — Additional file 1. [file 12882_2022_2906_MOESM1_ESM.docx]

**Supplementary table1.** The 143 differentially expressed genes information identified by differential gene expression analysis (58 were upregulated and 85 were downregulated in DN).

| **Symbol** | **log_2_FC** | **AveExpr** | **p.Value** | **adj.P.Val** | **Change** |
| --- | --- | --- | --- | --- | --- |
| DUSP1 | -1.25617 | 5.29114 | 3.76E-23 | 4.01E-19 | down |
| FOS | -2.6175 | 4.872139 | 4.56E-20 | 2.44E-16 | down |
| TGFBI | 1.542813 | 5.209413 | 1.14E-18 | 3.06E-15 | up |
| G6PC | -2.92274 | 4.707322 | 4.68E-18 | 9.99E-15 | down |
| ZFP36 | -2.02578 | 6.488748 | 3.35E-17 | 5.1E-14 | down |
| PRKAR2B | -1.43332 | 6.333434 | 4.26E-16 | 5.06E-13 | down |
| C1orf21 | -1.14408 | 4.950416 | 5.25E-16 | 5.61E-13 | down |
| COL1A2 | 2.025326 | 5.13844 | 1.24E-15 | 9.49E-13 | up |
| PDK4 | -1.83224 | 5.740396 | 5.58E-15 | 3.3E-12 | down |
| PLK2 | 1.206103 | 5.42454 | 8.49E-15 | 4.12E-12 | up |
| COL15A1 | 1.446617 | 4.378624 | 9.28E-15 | 4.31E-12 | up |
| LPL | -1.42772 | 4.903369 | 1.16E-14 | 5.14E-12 | down |
| CA10 | -1.33274 | 5.008615 | 1.2E-14 | 5.14E-12 | down |
| ALB | -2.44695 | 4.936927 | 1.48E-14 | 5.86E-12 | down |
| LRRC2 | -1.61007 | 5.794809 | 2.79E-14 | 9.94E-12 | down |
| HSD17B14 | -1.15575 | 4.565379 | 4.61E-14 | 1.54E-11 | down |
| DPP6 | -1.47731 | 6.028167 | 4.85E-14 | 1.57E-11 | down |
| JUNB | -1.42327 | 4.849406 | 7.82E-14 | 2.34E-11 | down |
| NPHS1 | -1.68593 | 6.711204 | 7.88E-14 | 2.34E-11 | down |
| CDH10 | -1.29729 | 3.687013 | 9.28E-14 | 2.61E-11 | down |
| IGFBP6 | 1.239681 | 5.188632 | 1.17E-13 | 3.05E-11 | up |
| G0S2 | -1.13317 | 6.122249 | 1.61E-13 | 3.76E-11 | down |
| CCDC91 | -1.03816 | 6.737839 | 1.62E-13 | 3.76E-11 | down |
| LUM | 2.673499 | 5.665852 | 2.12E-13 | 4.72E-11 | up |
| EGF | -1.48648 | 4.120973 | 2.56E-13 | 5.04E-11 | down |
| GPR18 | 1.048852 | 3.723417 | 2.58E-13 | 5.04E-11 | up |
| TPPP3 | -1.46654 | 6.667415 | 2.6E-13 | 5.04E-11 | down |
| COL6A3 | 1.808742 | 4.569743 | 6.48E-13 | 1.08E-10 | up |
| ARHGAP19 | -1.17189 | 6.454672 | 8.21E-13 | 1.31E-10 | down |
| S100A12 | -1.80937 | 4.122787 | 1.31E-12 | 2E-10 | down |
| TGFBR3 | -1.17331 | 8.025915 | 2.4E-12 | 3.37E-10 | down |
| RASL11B | -1.07991 | 5.636485 | 5.17E-12 | 6.5E-10 | down |
| MMP2 | 1.200659 | 4.856786 | 6.22E-12 | 7.55E-10 | up |
| THBS2 | 1.491578 | 4.540541 | 9.03E-12 | 1.05E-09 | up |
| TNNT2 | -1.42551 | 5.99021 | 1.01E-11 | 1.15E-09 | down |
| HSPA1B | -1.02645 | 6.566984 | 2.15E-11 | 2.03E-09 | down |
| C1QA | 1.130734 | 4.138224 | 2.23E-11 | 2.05E-09 | up |
| CA2 | -1.12765 | 6.241922 | 2.48E-11 | 2.23E-09 | down |
| FN1 | 2.474881 | 6.107393 | 3.56E-11 | 2.96E-09 | up |
| MPP5 | -1.00847 | 7.602476 | 4.21E-11 | 3.43E-09 | down |
| FCGR3B | -1.0495 | 5.299019 | 6.07E-11 | 4.6E-09 | down |
| TNC | 1.428057 | 5.842554 | 7.17E-11 | 5.32E-09 | up |
| TNNI1 | -1.40194 | 6.369125 | 7.5E-11 | 5.45E-09 | down |
| S100A9 | -1.76259 | 6.048858 | 8.68E-11 | 6.04E-09 | down |
| ADH1B | 1.669376 | 4.647273 | 8.75E-11 | 6.04E-09 | up |
| PTGDS | -1.22379 | 7.56569 | 9.57E-11 | 6.55E-09 | down |
| MS4A6A | 1.559153 | 5.191327 | 1.1E-10 | 7.43E-09 | up |
| MARCKS | 1.3835 | 5.724599 | 1.37E-10 | 8.73E-09 | up |
| S100A8 | -1.21098 | 5.389155 | 1.4E-10 | 8.91E-09 | down |
| TCF21 | -1.28322 | 8.502994 | 1.49E-10 | 9.36E-09 | down |
| VSIG4 | 1.375267 | 4.687881 | 1.62E-10 | 9.87E-09 | up |
| ARL4C | 1.163531 | 4.328373 | 1.72E-10 | 1.02E-08 | up |
| CYP27B1 | -1.10051 | 3.983412 | 2.04E-10 | 1.18E-08 | down |
| RGS2 | -1.38687 | 6.744093 | 2.15E-10 | 1.21E-08 | down |
| C1QB | 1.283892 | 4.307848 | 2.7E-10 | 1.48E-08 | up |
| EGR1 | -1.85617 | 6.229183 | 2.9E-10 | 1.55E-08 | down |
| DACH1 | -1.13668 | 6.931902 | 3.21E-10 | 1.69E-08 | down |
| AEBP1 | 1.168364 | 5.510497 | 3.94E-10 | 1.98E-08 | up |
| PCOLCE2 | -1.28255 | 7.754956 | 4.31E-10 | 2.12E-08 | down |
| PCK1 | -1.50715 | 6.092161 | 4.73E-10 | 2.3E-08 | down |
| SERPINF1 | 1.368193 | 4.331521 | 5.46E-10 | 2.61E-08 | up |
| FBP1 | -1.38107 | 5.865993 | 6.11E-10 | 2.86E-08 | down |
| ASPN | 1.240646 | 4.460783 | 1.33E-09 | 5.55E-08 | up |
| MAGI2 | -1.12626 | 5.715278 | 1.33E-09 | 5.55E-08 | down |
| ASPA | -1.02547 | 5.489977 | 1.62E-09 | 6.49E-08 | down |
| DCXR | -1.22266 | 7.222597 | 1.85E-09 | 7.23E-08 | down |
| ATF3 | -1.14756 | 4.791505 | 2.02E-09 | 7.74E-08 | down |
| DDN | -1.07436 | 5.819561 | 2.34E-09 | 8.57E-08 | down |
| CPA3 | 1.380514 | 3.877106 | 2.38E-09 | 8.66E-08 | up |
| MS4A4A | 1.316996 | 4.132236 | 2.38E-09 | 8.66E-08 | up |
| EMP1 | 1.006035 | 6.079152 | 3.06E-09 | 1.06E-07 | up |
| HPD | -1.9221 | 6.024437 | 3.34E-09 | 1.14E-07 | down |
| TAGLN | 1.062201 | 5.493253 | 4.41E-09 | 1.47E-07 | up |
| FMO3 | 1.245384 | 4.071413 | 4.46E-09 | 1.47E-07 | up |
| FOSB | -1.23444 | 3.666511 | 5.82E-09 | 1.83E-07 | down |
| GSTA1 | -1.82405 | 7.735812 | 6.55E-09 | 2E-07 | down |
| ALDH6A1 | -1.19146 | 6.479679 | 7.5E-09 | 2.25E-07 | down |
| PTN | 1.23056 | 5.031244 | 8.77E-09 | 2.57E-07 | up |
| EDNRB | 1.033751 | 5.298369 | 1.08E-08 | 3.1E-07 | up |
| FABP1 | -1.32808 | 5.402872 | 1.34E-08 | 3.64E-07 | down |
| AFM | -1.4822 | 4.823626 | 1.72E-08 | 4.42E-07 | down |
| SLC7A7 | -1.02754 | 6.463113 | 1.81E-08 | 4.58E-07 | down |
| RARRES1 | 1.254738 | 3.832766 | 1.97E-08 | 4.92E-07 | up |
| CD44 | 1.120641 | 4.956919 | 2.23E-08 | 5.44E-07 | up |
| PRODH2 | -1.2079 | 5.912234 | 2.33E-08 | 5.62E-07 | down |
| PTGS2 | -1.29458 | 3.576325 | 2.47E-08 | 5.9E-07 | down |
| RGS5 | 1.184934 | 5.270243 | 2.48E-08 | 5.9E-07 | up |
| HAO2 | -1.70489 | 5.667976 | 2.63E-08 | 6.2E-07 | down |
| EHD3 | -1.36058 | 7.553206 | 2.82E-08 | 6.58E-07 | down |
| SEMA3G | -1.02136 | 6.418161 | 4.06E-08 | 8.91E-07 | down |
| CD53 | 1.131428 | 5.999391 | 5.48E-08 | 1.14E-06 | up |
| C7 | 1.677341 | 6.920104 | 5.8E-08 | 1.19E-06 | up |
| CHI3L1 | -1.2667 | 7.950579 | 7.13E-08 | 1.4E-06 | down |
| MT1G | -1.02924 | 9.813623 | 9.06E-08 | 1.71E-06 | down |
| GLYAT | -1.58081 | 6.593434 | 9.29E-08 | 1.74E-06 | down |
| CD163 | 1.228452 | 4.681327 | 9.85E-08 | 1.83E-06 | up |
| C3 | 1.853259 | 5.718639 | 1.22E-07 | 2.17E-06 | up |
| ECM1 | 1.211484 | 6.588534 | 1.27E-07 | 2.23E-06 | up |
| KRT19 | 1.292327 | 5.228405 | 1.5E-07 | 2.56E-06 | up |
| CLU | 1.024238 | 5.609941 | 1.62E-07 | 2.7E-06 | up |
| COL3A1 | 1.136714 | 5.507848 | 1.64E-07 | 2.73E-06 | up |
| KLK7 | -1.04276 | 7.213136 | 1.98E-07 | 3.22E-06 | down |
| NR4A2 | -1.06738 | 3.598324 | 2.02E-07 | 3.26E-06 | down |
| FBLN5 | 1.188566 | 5.761227 | 2.31E-07 | 3.63E-06 | up |
| ZNF804A | -1.07708 | 4.977757 | 2.62E-07 | 4.04E-06 | down |
| CD52 | 1.067956 | 5.22231 | 2.78E-07 | 4.23E-06 | up |
| CCL21 | 1.677084 | 4.674615 | 3.55E-07 | 5.17E-06 | up |
| POSTN | 1.022499 | 7.686635 | 3.62E-07 | 5.25E-06 | up |
| CCL19 | 1.639328 | 4.41691 | 3.7E-07 | 5.35E-06 | up |
| BHMT | -1.40694 | 7.457539 | 3.79E-07 | 5.47E-06 | down |
| ALDOB | -1.52658 | 9.44285 | 4.34E-07 | 6.11E-06 | down |
| HPGD | -1.22205 | 5.956186 | 5.15E-07 | 7.01E-06 | down |
| APOH | -1.00428 | 3.911685 | 6.2E-07 | 8.14E-06 | down |
| SERPINA3 | 1.029133 | 4.014598 | 6.98E-07 | 9E-06 | up |
| CDH6 | 1.216635 | 4.793499 | 1.37E-06 | 1.57E-05 | up |
| VCAN | 1.564112 | 5.006537 | 1.69E-06 | 1.85E-05 | up |
| IGF1 | -1.53723 | 5.783756 | 1.78E-06 | 1.91E-05 | down |
| TNNC1 | -1.12315 | 5.793674 | 2.33E-06 | 2.39E-05 | down |
| RNASE6 | 1.331773 | 5.208479 | 3.13E-06 | 3.03E-05 | up |
| B3GALT2 | -1.19631 | 5.018884 | 3.28E-06 | 3.15E-05 | down |
| CALB1 | -1.35268 | 5.213043 | 3.96E-06 | 3.7E-05 | down |
| ANGPTL3 | -1.0328 | 4.436518 | 5.59E-06 | 4.94E-05 | down |
| IGKC | 1.479824 | 6.022183 | 7.28E-06 | 6.13E-05 | up |
| FPR3 | 1.05419 | 4.105281 | 7.55E-06 | 6.28E-05 | up |
| BHMT2 | -1.00504 | 6.854942 | 8.01E-06 | 6.61E-05 | down |
| SST | -1.20248 | 4.742074 | 9.28E-06 | 7.43E-05 | down |
| GATM | -1.14605 | 7.959612 | 1.35E-05 | 0.000101 | down |
| UMOD | -1.68278 | 6.745611 | 1.67E-05 | 0.000121 | down |
| MMP7 | 1.588765 | 7.200078 | 3.21E-05 | 0.000209 | up |
| SLC7A9 | -1.01886 | 5.682788 | 3.43E-05 | 0.000221 | down |
| APOM | -1.14888 | 6.2202 | 3.6E-05 | 0.00023 | down |
| PLCG2 | -1.15174 | 5.943359 | 3.85E-05 | 0.000244 | down |
| SLC12A3 | -1.12913 | 4.721844 | 4.17E-05 | 0.000261 | down |
| CCL18 | 1.302816 | 3.269119 | 4.35E-05 | 0.000271 | up |
| LTF | 1.032316 | 5.058489 | 7.65E-05 | 0.000442 | up |
| CYP4A11 | -1.10799 | 5.348524 | 7.66E-05 | 0.000442 | down |
| APOC1 | 1.254343 | 5.355056 | 8.15E-05 | 0.000466 | up |
| DEFB1 | -1.02161 | 8.8999 | 0.000103 | 0.000564 | down |
| KNG1 | -1.25713 | 6.662678 | 0.000115 | 0.000624 | down |
| COLEC12 | 1.180747 | 4.907274 | 0.000159 | 0.000817 | up |
| XPNPEP2 | -1.02238 | 5.481344 | 0.000201 | 0.000991 | down |
| IGHM | 1.00147 | 4.483884 | 0.006743 | 0.019181 | up |
| REN | 1.066683 | 7.975296 | 0.010518 | 0.027835 | up |
